# 类红细胞状表面分子印迹聚合物的制备及其用于牛奶中四环素的选择性富集

**DOI:** 10.3724/SP.J.1123.2024.06008

**Published:** 2024-12-08

**Authors:** Jian SHANG, Linzhi MA, Chao LEI, Xinke SU, Yu LIU, Yue WANG, Ruixia GAO

**Affiliations:** 1.渭南市检验检测研究院, 陕西 渭南 714000; 1. Weinan Inspection and Research Institute, Weinan 714000, China; 2.西安交通大学化学学院, 陕西 西安 710049; 2. School of Chemistry, Xi’an Jiaotong University, Xi’an 710049, China; 3.西安交通大学药学院, 陕西 西安 710061; 3. School of Pharmacy, Xi’an Jiaotong University, Xi’an 710061, China

**Keywords:** 类红细胞状分子印迹聚合物, 表面印迹, 四环素, 选择性吸附, red-blood-cell-like surface molecularly imprinted polymer (RBC-SMIPs), surface imprinting, tetracycline (TC), selective adsorption

## Abstract

分子印迹聚合物单位质量吸附容量是评价印迹材料吸附性能的重要指标之一,受红细胞双凹圆碟形貌比表面积大和优异稳定性的启发,合成了对四环素具有高选择性、大吸附容量、较快传质速率的类红细胞状表面分子印迹聚合物(RBC-SMIPs)。本工作首先以SiO_2_作为初始载体,广谱抗生素四环素作为模板分子,通过表面分子印迹技术将廉价易得的多巴胺功能单体包覆在SiO_2_载体表面,然后通过牺牲载体刻蚀技术将SiO_2_初始载体刻蚀去除,并调节多巴胺印迹层用量和包覆时间,调控所制备印迹材料的形貌,最终制备出大比表面积和优异选择性吸附性能的RBC-SMIPs。通过扫描电镜(SEM)、红外光谱(FT-IR)、N_2_吸附-解吸附等表征手段以及动力学、热力学、选择性、重复利用性等吸附实验证明该材料成功包覆聚多巴胺印迹层,所制备材料粒径均一、比表面积大、传质速率快(15 min)、重复利用性好、单位质量吸附容量和印迹因子分别高达76.16 mg/g和3.62。此外,将RBC-SMIPs作为固相萃取吸附剂与高效液相色谱结合,该方法在0.01~200 μg/mL范围内具有较高的线性相关系数(*R*^2^>0.9995),在3个加标水平下(0.01、0.05和0.10 μg/mL)平均回收率为92.1%~95.3%,实现了对牛奶样品中四环素的高效吸附、富集和检测。本工作所开发的类红细胞状表面分子印迹聚合物具有良好的实际应用能力。

## Abstract (en)

The adsorption capacity per unit mass of a molecularly imprinted polymer is an important indicator for evaluating the adsorption performance of an imprinted material. Inspired by the large specific surface areas and excellent stabilities of double concave circular red-blood-cell discs, we synthesized a highly selective red-blood-cell-like surface molecularly imprinted polymer (RBC-SMIP) with a large adsorption capacity and a fast mass-transfer rate for tetracycline. This work used SiO_2_ as the initial carrier and tetracycline, a broad-spectrum antibiotic, as the template molecule. Inexpensive and readily available dopamine functional monomers were subsequently coated on the surface of the SiO_2_ carrier using surface molecular imprinting technology, after which the initial SiO_2_ carrier was etched and removed using sacrificial-carrier-etching technology, with the dosage and coating time of the dopamine imprinting layer adjusted to regulate the morphology of the prepared imprinted material. An RBC-SMIP with a large specific surface area and excellent selective adsorption performance was finally prepared. Scanning electron microscopy (SEM) and infrared (FT-IR) spectroscopy, as well as N_2_-adsorption-desorption, kinetics, thermodynamics, selectivity, and reusability experiments confirmed that the material had successfully been coated the imprinted polydopamine layer, and that the prepared material contained particles of uniform size and exhibited a good specific surface area and pore volume, a rapid adsorption-equilibrium time (15 min), and excellent reusability. The RBC-SMIP exhibited a unit-mass adsorption capacity and imprinting factor of 76.16 mg/g and 3.62, respectively. The successful preparation of the RBC-SMIP provides a new approach for the selective adsorption and determination of trace tetracycline contents in environmental water. In addition, combining the RBC-SMIP (as a solid-phase extractor) with HPLC led to a method with a high linear correlation coefficient (*R*^2^>0.9995) in the 0.01-200 μg/mL linear range. Average recoveries of 92.1%-95.3% were determined at three spiked levels (0.01, 0.05, and 0.10 μg/mL), highlighting the ability of the method to efficiently adsorb, enrich, and detect tetracycline in milk samples. The red-blood-cell-like surface molecularly imprinted polymer developed in this work is expected to have significant practical applications.

四环素(TC)作为广谱型抗生素,对革兰氏阴性细菌、革兰氏阳性细菌、螺旋体、钩端螺旋体、大型病毒及原生动物均表现出活性。因价格低廉、亲水性优良、低挥发性和长半衰期^[1]^,TC广泛应用于临床医学、畜牧和水产养殖等领域^[2]^。然而,TC的过度使用,导致其在动物源性食品中微量残留。牛奶作为一种常见的动物源性食品,其TC残留量已引起世界各地食品安全和检验机构的广泛关注。目前,欧盟、美国食品法典委员会、中国农业部将牛奶中TC残留量严格限制在0.100 μg/mL以下^[3]^。2019年,我国发布的食品安全国家标准(GB 31650-2019)^[4]^规定牛奶中TC残留量不得超过100 μg/kg。尽管牛奶中残留TC含量很低,但其通过食物链不断在人体内富集,不仅会导致关节疾病、神经病变、内分泌紊乱,还可能导致人体产生耐药性和过敏反应,危害人体健康^[5⇓-7]^。因此,开发一种高效的分析方法用于检测和去除动物源性食品中的痕量TC具有重要意义。

近年来,检测TC的方法包括电化学分析法^[8]^、表面增强拉曼光谱法^[9]^、荧光分析法^[10]^、高效液相色谱法^[11]^等。然而,食品样品基质复杂,TC含量较低,不易被直接检测。样品预处理是对TC进行富集和减轻基质干扰的必要前提条件,一般的样品前处理方法有固相萃取^[12]^、液液萃取^[13]^、基质辅助固相萃取^[14]^等。其中,SPE因操作便捷、有机溶剂使用量少、便于自动化等优势而被广泛使用。固相萃取吸附剂是决定固相萃取效率和选择性的关键,传统固相萃取吸附剂包括碳纳米管、金属有机框架材料和二氧化硅等^[15,16]^,但这些吸附剂对分析物往往不具有选择性^[17]^。为解决上述问题,设计一种对分析物具有高选择性的固相萃取吸附剂具有重要意义。

表面分子印迹聚合物(SMIPs)是指模板分子和功能单体通过共价或非共价形式结合,模板分子被移除后聚合物印迹层形成大量均一、有序的印迹位点,可极大提高材料对分析物的吸附容量和选择性^[18,19]^。Lv等^[20]^采用甲基丙烯酸-二氧化硅杂化复合材料用于牛奶中四环素类残留物的选择性萃取,其吸附容量可达46.65 mg/g。Liu等^[21]^利用多巴胺的自聚合将其包覆在石墨烯表面形成SMIPs,成功应用于牛奶样品中TC的检测,最大吸附容量为21.28~40.43 μg/g。Niu等^[22]^基于改性的二氧化硅制备SMIPs,用于分析牛奶样品中残留的TC,其吸附容量为42.30 mg/g。Li等^[23]^以Fe_3_O_4_为磁芯,以多孔纤维素为载体,开发了一种磁性多孔纤维素表面分子印迹聚合物,用于对环境水、牛肉和牛奶样品中TC的去除和检测,最大吸附容量为14.50 mg/g。虽然这些材料提高了对四环素的选择性吸附能力,但不具有识别位点的载体占据材料的大部分质量,从而大大降低单位质量印迹聚合物的吸附容量。受红细胞双凹圆碟状形貌具有较大比表面积和优异悬浮稳定性的启发,设计了一种载体质量较轻、比表面积较大的SMIPs,增加其与模板分子的接触率,从而提高材料对模板分子的吸附性能,以实现对模板分子的高效吸附、富集和检测。

本工作以SiO_2_为初始载体、TC为模板分子、多巴胺(DA)为功能单体,利用表面分子印迹技术和牺牲载体刻蚀技术,提出了一种具有均匀印迹位点的类红细胞状表面分子印迹聚合物(RBC-SMIPs)。DA具有官能团丰富、黏附性强、价格低廉、刚性好等优点,被认为是一种理想的功能单体。DA包覆于载体表面后,通过刻蚀手段牺牲SiO_2_,制备得到类红细胞状表面分子印迹聚合物。所制备印迹材料与普通球形结构表面印迹材料相比,具有较大比表面积,增加了吸附位点与目标物的接触面积,大大提高了材料单位质量吸附容量和传质速率。

## 1 实验部分

### 1.1 仪器与试剂

LCMS-2020超高效液相色谱-四极杆质谱仪(西安杰森科学发展有限公司), PHS-3C电子天平(杭州奥立龙仪器有限公司), VERTEX70场发射扫描电子显微镜(上海卡尔察司管理有限公司), AWL-0502-U艾科浦超纯水机(重庆颐洋企业发展有限公司), VERTEX70在线红外光谱仪(陕西盈美电子科技有限公司), KQ-100E超声波清洗器(昆山市超声仪器有限公司), RW20搅拌器(德国艾卡公司), DL-101电热恒温鼓风干燥箱(天津市中环实验电炉有限公司), HY-2调速多用振荡器(上海强运科技有限公司), UV-1800紫外-可见分光光度计(日本岛津公司), ASAP 2020 Plus HD88全自动物理吸附仪(陕西朗润国际贸易有限公司)。

氨水(NH_3_·H_2_O)、乙醇、乙酸、正硅酸乙酯(TEOS)、三羟甲基氨基甲烷盐酸盐(Tris-HCl)和碳酸钠(Na_2_CO_3_)均为分析纯,甲醇和乙腈为色谱纯,购于上海远帆生物科技有限公司。TC、土霉素(OTC)、强力霉素(DC)、金霉素(CTC)和氯霉素(CAP)、DA均为分析纯,购于北京迈瑞达科技有限公司。实验用水为超纯水。牛奶样品购自陕西西安市超市。

### 1.2 SiO_2_球的制备

将16.25 mL乙醇、24.75 mL超纯水、9 mL氨水混合在250 mL三口瓶中,在上述溶液中加入4.5 mL TEOS,室温搅拌2 h,用超纯水和无水乙醇反复洗涤后离心(10000 r/min)10 min,分离得到SiO_2_球。

### 1.3 RBC-SMIPs的制备

首先,在250 mL的三口瓶中加入100 mg SiO_2_、30 mg TC、40 mL Tris-HCl(10 mmol/L, pH=8.5)和20 mL乙醇,室温搅拌30 min。随后,向上述三口瓶中加入80 mg DA,混合物在室温下搅拌7 h。最后,制备的纳米材料用超纯水和无水乙醇交替洗3遍后离心(10000 r/min)10 min收集。

取250 mg制备的纳米材料,加入到50 mL Na_2_CO_3_(0.6 mol/L)溶液中,在80 ℃下搅拌1 h,刻蚀去除SiO_2_,得到类红细胞状纳米材料。最后,采用乙醇-乙酸(99∶1, v/v)洗脱去除模板分子TC,制备得到RBC-SMIPs,然后对溶液离心(10000 r/min)10 min,并用超纯水和无水乙醇交替洗3遍,40 ℃烘干至恒重。

类红细胞状非表面分子印迹聚合物(RBC-SNIPs)的制备除不添加模板分子TC以外,其余步骤同上。

### 1.4 材料的吸附性能考察

#### 1.4.1 动力学吸附性能

取10 mg RBC-SMIPs或RBC-SNIPs放入10 mL 250 μg/mL TC溶液中,在180 r/min转速的振荡器中室温振荡5、10、15、20、25、30 min后,离心(10000 r/min)10 min,将吸附材料与溶液分离,用紫外可见分光光度计测定上清液吸光度。利用公式(1)对TC的吸附量进行计算。


(1) $$Q=\frac{\left(C_{0}-C_{e}\right)V}{W}$$

其中,*Q*(mg/g)是材料吸附量,*C*_0_(μg/mL)表示TC溶液初始质量浓度,*C*_e_(μg/mL)表示吸附达平衡时TC的质量浓度,*V*(mL)代表溶液体积,*W*(mg)是材料质量。

#### 1.4.2 等温吸附性能

取10 mg的RBC-SMIPs或RBC-SNIPs放入10 mL不同质量浓度(50、100、150、200、250、300和350 μg/mL)的TC溶液中,在180 r/min转速的振荡器中室温振荡15 min后,离心(10000 r/min)10 min,将吸附材料与溶液分离,用紫外可见分光光度计测定上清液的吸光度。

#### 1.4.3 选择性吸附性能

取10 mg的RBC-SMIPs或RBC-SNIPs分别放入10 mL质量浓度均为250 μg/mL的TC、OTC、DC、CTC、CAP的单一溶液中,在180 r/min转速的振荡器中室温振荡15 min后,离心(10000 r/min)10 min,将吸附材料与溶液分离,上清液中的四环素类抗生素和CAP的浓度用紫外可见分光光度计测定。印迹因子(IF)和选择性因子(SC)通常用来评估印迹材料对模板分子的选择性性能,其计算公式如下。


(2) $$IF=\frac{Q_{SMIP}}{Q_{SNIP}}$$

(3) $$SC=\frac{{IF}_{TEM}}{{IF}_{COM}}$$

其中,*Q*_SMIPs_和*Q*_SNIPs_(mg/g)分别代表RBC-SMIPs和RBC-SNIPs对模板分子TC的吸附量;IF_TEM_与IF_COM_分别表示材料对TC和其他竞争物的印迹因子。

### 1.5 牛奶样品分析

取10 mL牛奶样品(经检测为阴性)放入离心管中,加入3种不同质量浓度(0.01、0.05和0.10 μg/mL)的TC标准溶液,再与20 mL甲醇混合,振摇20 min,使生物大分子蛋白质等沉淀。然后离心(10000 r/min)10 min,用0.22 μm滤膜过滤上清液。随后,将10 mg RBC-SMIPs加入上述加标牛奶样品中,在室温下振荡30 min,离心(10000 r/min)10 min后分离纳米材料,并用乙醇-乙酸(99∶1, v/v)对材料进行洗脱。收集洗脱液并在氮气流下蒸发至干燥。最后,将洗脱的剩余物溶于0.5 mL甲醇,用于HPLC检测。

### 1.6 分析条件

色谱柱为YMC-Triart C18柱(150 mm×4.6 mm, 5 μm),柱温为40 ℃,检测波长为357 nm,流动相为乙腈-0.05%乙酸溶液(60∶40, v/v),流速为0.5 mL/min,进样量为20 μL。

## 2 结果与讨论

### 2.1 RBC-SMIPs的制备

本工作通过将表面分子印迹技术和牺牲载体方法相结合,合成RBC-SMIPs(如图1所示)。首先,采用改进的Stöber法制备具有丰富羟基的SiO_2_并将其作为牺牲载体。随后,以含有邻苯二酚和氨基的DA为功能单体、TC为模板分子、SiO_2_为载体,基于DA在弱碱性条件下自聚合的原理,通过氢键和*π-π*相互作用将模板TC固定在SiO_2_表面。使用Na_2_CO_3_完全刻蚀SiO_2_使聚合物质量大大降低,刻蚀后的材料为类似于红细胞的双凹圆碟状。最后,通过乙醇-乙酸(99∶1, v/v)洗脱液破坏TC和功能单体的相互作用,从而得到RBC-SMIPs。

**图 1:**
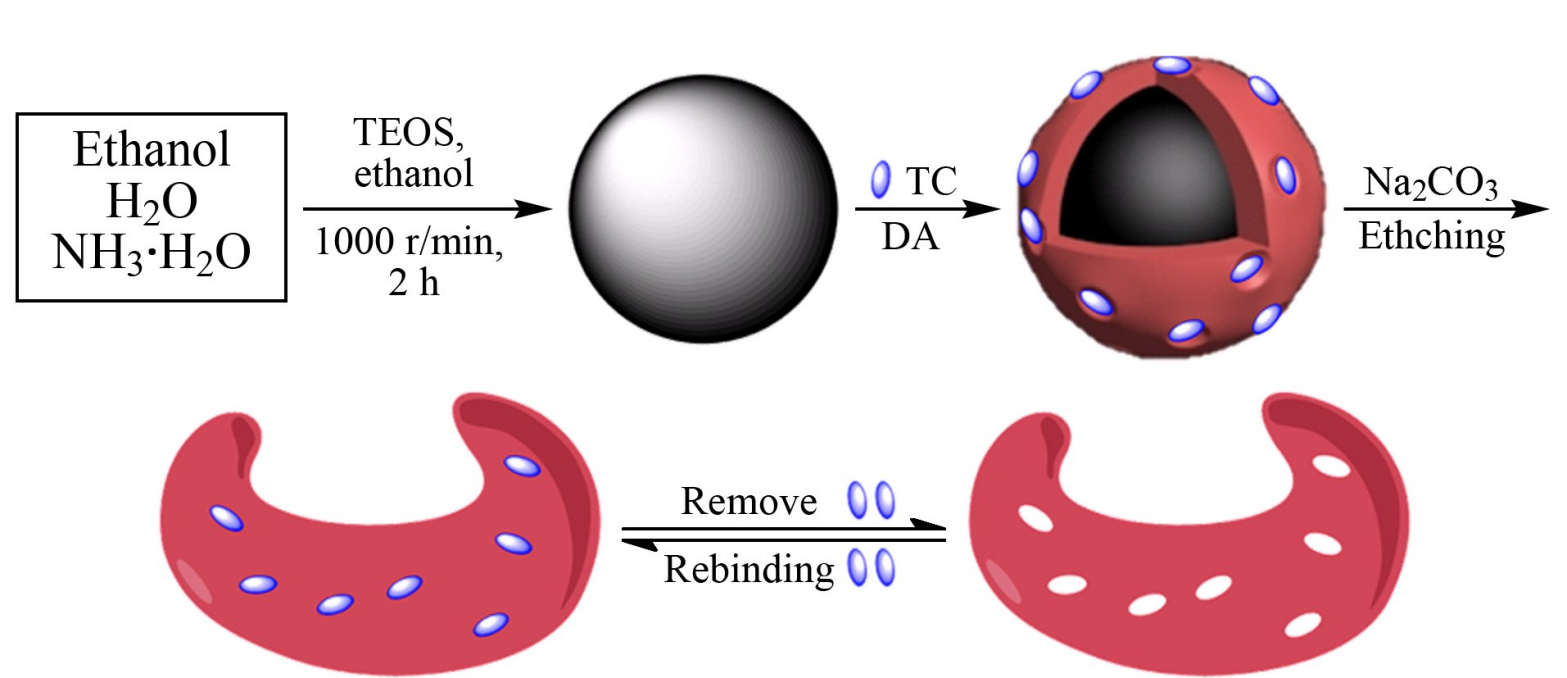
类红细胞状表面分子印迹聚合物的制备过程

### 2.2 聚合条件的考察

为获得更多的特异性识别位点和良好的吸附能力,对DA的包覆用量以及聚合时间进行考察,从而获得最佳的印迹效果。

在载体SiO_2_用量(100 mg)不变的条件下,选取DA用量为60、70、80、90、100 mg进行考察,如图2a显示,随着印迹层DA用量的增加,吸附量和印迹因子呈现先增大后减小的趋势,当DA用量小于80 mg时,由于印迹层的厚度增加使特异性吸附位点数量增加,在80 mg时*Q*和IF最大;随着DA用量继续增加,*Q*和IF逐渐下降,可能是由于多余的DA发生自聚或印迹层过厚使得包覆在内部的模板分子TC不易完全洗脱,从而造成模板泄露并阻碍吸附传质过程。根据上述实验数据可知,确定DA 80 mg为最佳用量。

**图 2:**
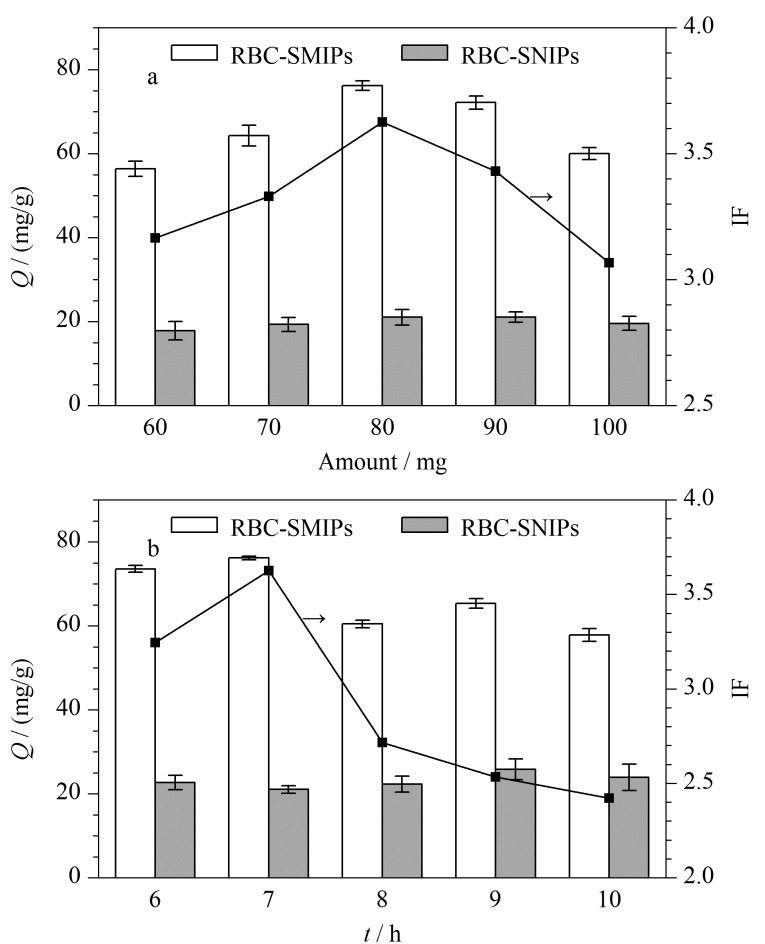
(a)DA用量和(b)聚合时间对RBC-SMIPs和RBC-SNIPs吸附TC效果的影响(*n*=3)

在确定DA用量为80 mg的前提下,选取6、7、8、9、10 h聚合时间进行考察(图2b)。聚合时间考察与包覆用量考察显示出同样趋势,当反应时间为7 h时,RBC-SMIPs显示出最高的吸附量和最高的印迹因子,可能是因为在此时间条件下可形成与模板分子结合的最合适印迹位点数量以及适当印迹层厚度,当聚合反应时间大于7 h时,*Q*和IF有所减小,主要是因为聚合时间过长导致印迹位点包覆过深,在吸附过程中模板分子不易到达。根据上述实验数据可知,最佳聚合时间为7 h。

### 2.3 材料表征

#### 2.3.1 扫描电镜分析

图3为SiO_2_、SiO_2_@DA和RBC-SMIPs的SEM图。可以看出,SiO_2_(图3a)呈形状规则的球形,表面光滑,粒径均一,分散度良好,直径约为400 nm,经过DA包覆后(图3b),直径较SiO_2_更大,材料表面变得粗糙,可以明显看到印迹层存在;经过Na_2_CO_3_刻蚀后,材料呈现类红细胞状两面凹圆碟状(图3c),证明刻蚀材料成功合成,该结构具有较大的比表面积和丰富的吸附位点,较普通球形材料具有更好的传质能力,更有利于吸附过程进行。

**图 3:**
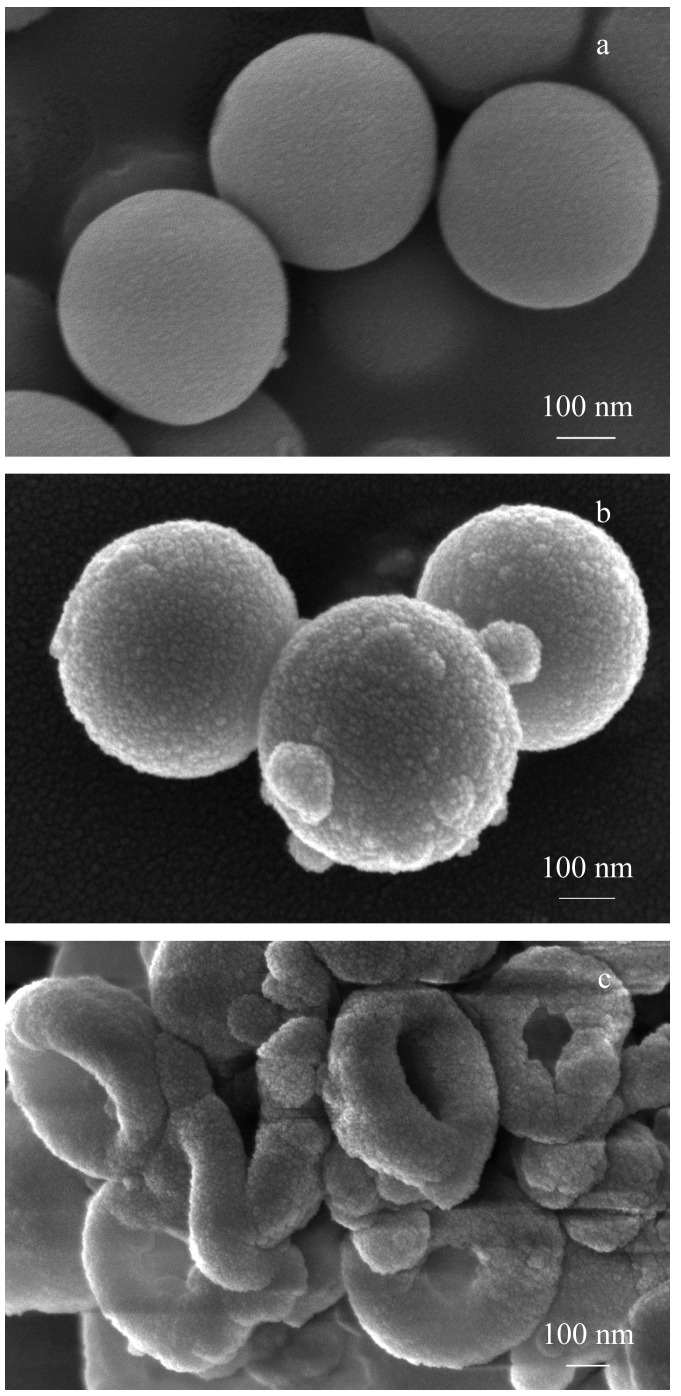
(a)SiO_2_、(b)SiO_2_@DA和(c)RBC-SMIPs的扫描电镜图

#### 2.3.2 红外光谱分析

SiO_2_、SiO_2_@DA和RBC-SMIPs的红外光谱如图4所示。

**图 4:**
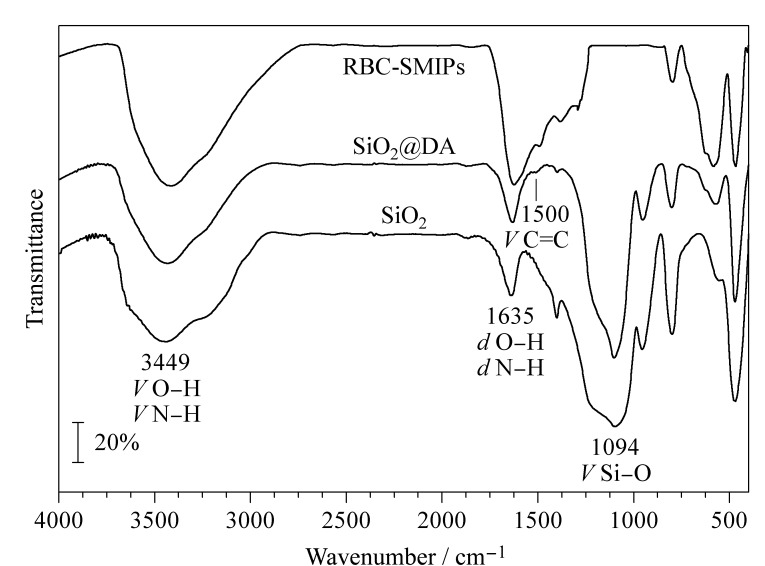
SiO_2_、SiO_2_@DA和RBC-SMIPs的红外光谱图

SiO_2_、SiO_2_@DA和RBC-SMIPs在1635 cm^-1^和3449 cm^-1^处的两个特征峰都表示O-H和N-H的弯曲和伸缩振动特性,SiO_2_@DA和RBC-SMIPs在1500 cm^-1^处特征峰表示苯环结构C=C的伸缩振动,表明DA成功包覆在SiO_2_上,SiO_2_@DA成功制备。在SiO_2_和SiO_2_@DA中,在1094 cm^-1^处产生Si-O-Si的伸缩振动^[24]^,而RBC-SMIPs在1094 cm^-1^处失去伸缩振动,表明SiO_2_载体被成功刻蚀并且被刻蚀完全,证明RBC-SMIPs成功制备。

#### 2.3.3 N_2_吸附解吸曲线分析

图5a分别为SiO_2_@DA和RBC-SMIPs的氮气吸附和脱附实验测试结果,比表面积分别为12.59 m^2^/g和20.43 m^2^/g,由于Na_2_CO_3_刻蚀载体的作用,RBC-SMIPs的比表面积显著增加。图5b为SiO_2_@DA和RBC-SMIPs的孔隙体积,分别为0.0250 cm^3^/g和0.1291 cm^3^/g,相比于SiO_2_@DA, RBC-SMIPs总孔体积扩大约5倍。RBC-SMIPs与SiO_2_@DA相比,具有更大的比表面积和更高孔容,可显著提高基质扩散速度,增加吸附位点,有助于提高材料吸附容量。

**图 5:**
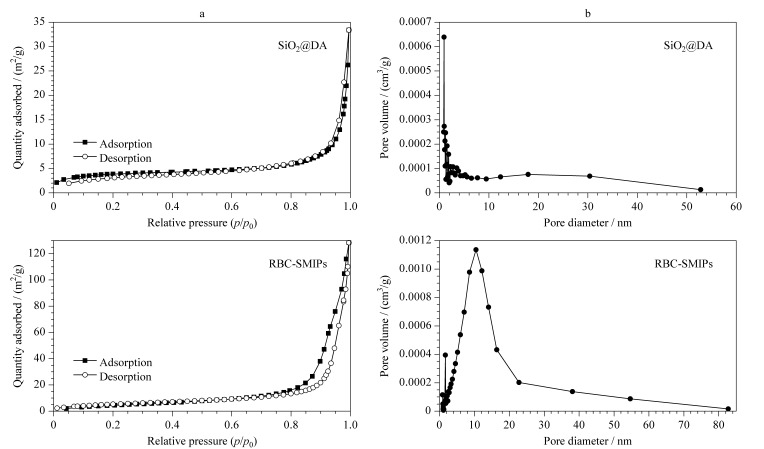
SiO_2_@DA和RBC-SMIPs的(a)氮气吸附-脱附等温线和(b)孔隙体积

### 2.4 材料吸附性能考察

#### 2.4.1 动力学和等温吸附性能考察

RBC-SMIPs和RBC-SNIPs的动力学吸附实验结果见图6a。对材料进行5~30 min的吸附,吸附时间增加后,吸附量也随之增加,这是因为随吸附时间增加,RBC-SMIPs上的印迹识别位点逐渐与模板分子TC结合。当吸附时间为15 min时,RBC-SMIPs和RBC-SNIPs的吸附量达到最大且几乎不再变化,这是因为材料上吸附位点与模板分子结合达到动态平衡,而RBC-SMIPs中具有大量的特异性识别位点,所以平衡时吸附量较RBC-SNIPs更高。图6b为RBC-SMIPs和RBC-SNIPs的等温吸附实验结果。将材料加入到50~350 μg/mL范围的TC溶液中,当TC质量浓度小于250 μg/mL时,材料吸附量随着浓度的增大而增大;当TC的质量浓度达到250 μg/mL时,材料吸附量达到最大,继续增大TC的质量浓度,吸附量几乎不变,这是因为此浓度下RBC-SMIPs中特异性和非特异性识别位点与模板分子结合均已达平衡(76.16 mg/g), RBC-SNIPs仅有非特异性吸附,在该浓度下吸附达到平衡,但吸附量较RBC-SMIPs低,为21.01 mg/g,材料IF为3.62。

**图 6:**
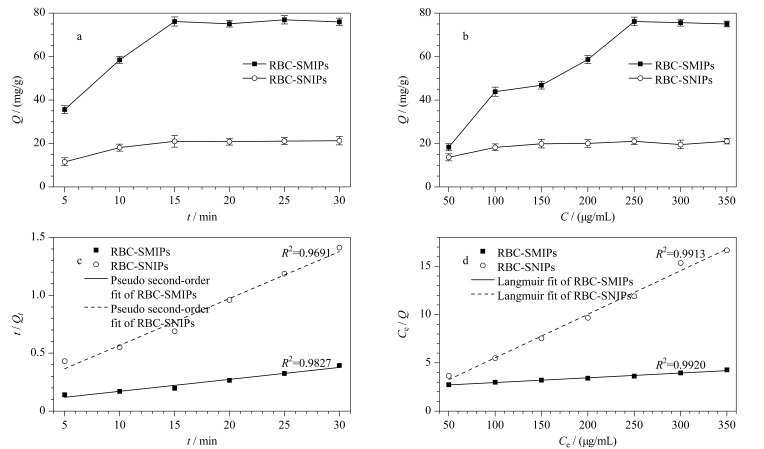
RBC-SMIPs和RBC-SNIPs的(a)动力学吸附图、(b)等温吸附图、(c)拟二级动力学拟合曲线以及(d)热力学Langmuir拟合曲线(*n*=3)

采用拟一级和拟二级动力学模型拟合动力学数据,结果表明,拟二级动力学模型(*R*^2^=0.9691)更符合实验数据(图6c)。这表示化学吸附可能是RBC-SMIPs和RBC-SNIPs吸附过程中的决速步骤^[25]^。

为进一步探究等温吸附过程,选择Freundlich和Langmuir等温模型对等温数据拟合,结果表明,Langmuir等温吸附模型(*R*^2^=0.9913)更符合实验数据(图6d),说明RBC-SMIPs和RBC-SNIPs对TC的吸附可能是单层吸附^[26]^。

#### 2.4.2 选择性吸附性能考察

本研究选择具有代表性的4种抗生素来评估RBC-SMIPs和RBC-SNIPs的特异性吸附能力,即以3种结构类似物四环素类抗生素(OTC、CTC、DC)以及共存物CAP作为竞争物。由图7可知,RBC-SMIPs对TC吸附量(76.16 mg/g)和印迹因子(3.62)均大于其他4种竞争物,对于CAP吸附量远低于TC,且RBC-SMIPs和RBC-SNIPs对于CAP的吸附容量相差较小,这是因为RBC-SMIPs和RBC-SNIPs对CAP的吸附都是非特异性吸附,CAP分子结构与TC差异性大,难以接近印迹空穴。OTC、CTC、DC作为四环素类抗生素,具有与TC高度相似的结构,因此RBC-SMIPs对于3种结构类似物也具有一定识别作用。

**图 7:**
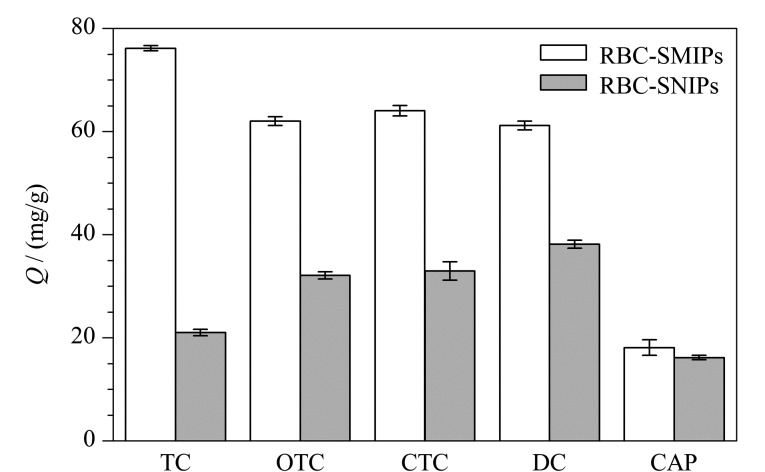
RBC-SMIPs和RBC-SNIPs对四环素及4种竞争物的选择性吸附性能考察(*n*=3)

#### 2.4.3 重复利用性能考察

为考察材料的重复利用性能,采用同一RBC-SMIPs和RBC-SNIPs进行8次吸附-解吸附实验(图8)。随着材料吸附-解吸附次数增加,有一部分印迹位点因被模板分子TC堵塞而难以洗脱,导致材料对TC吸附量轻微减小。当重复使用次数达到8次时,RBC-SMIPs的吸附量仍然可以达到第一次使用时吸附量的94.62%,说明材料具有良好的重复利用性。而RBC-SNIPs对TC的吸附属于非特异性吸附,几乎未受洗脱影响。

**图 8:**
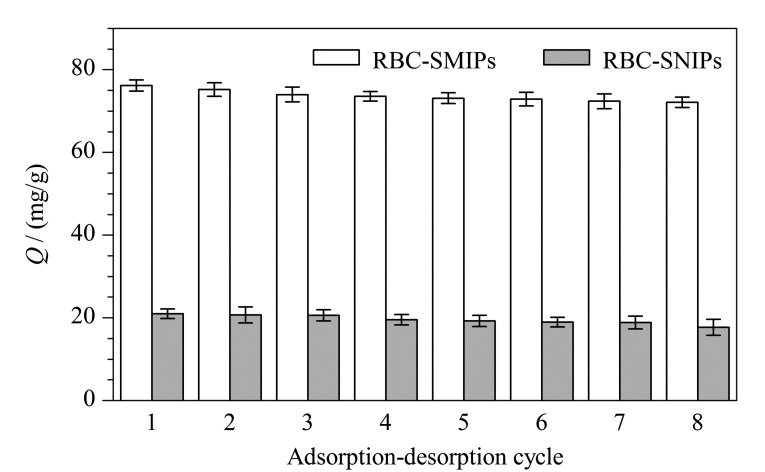
RBC-SMIPs和RBC-SNIPs的重复使用性(*n*=3)

#### 2.4.4 方法评估与实际样品分析

本工作建立了一种以RBC-SMIPs作为固相萃取剂结合HPLC高效富集和选择性检测牛奶中痕量TC的新方法。在TC质量浓度为0.01~200 μg/mL的线性范围内,具有较高的线性相关系数(*R*^2^>0.9995),按照3倍和10倍信噪比(*S/N*)计算检出限(LOD)和定量限(LOQ),分别为1.18 ng/mL和3.45 ng/mL。

为了评估RBC-SMIPs的实际应用性,将其作为固相萃取吸附剂,结合高效液相色谱,对加标不同质量浓度(0.01、0.05和0.10 μg/mL)TC的牛奶样品进行分析(*n*=3)。表1显示材料对加标牛奶中TC的回收率为92.1%~95.3%, RSD为0.3%~1.9%。实验结果表明,该方法较为灵敏且准确度较好。

**表 1:** 牛奶样品中TC在3个水平下的加标回收率(*n*=3)

Spiked/(μg/mL)	Detected/(μg/mL)	Recovery/%	RSD/%
0.01	0.00921	92.1	1.5
0.05	0.0468	93.2	0.3
0.10	0.0953	95.3	1.9

图9为TC标准溶液(图9a)、加标TC(0.0500 μg/mL)的牛奶(图9b)和吸附加标牛奶后RBC-SMIPs的洗脱富集(图9c)色谱图。如图9b所示,加标牛奶中未检测到TC,但有其他杂峰出现,表明牛奶为复杂基质。RBC-SMIPs吸附加标牛奶样后采用洗脱液乙醇-乙酸(99∶1, v/v)对RBC-SMIPs进行洗脱和氮气吹干,将剩余物用甲醇溶解,经分析在约6.0 min时出现尖锐清晰的TC吸收峰(图9c),这与TC标准品的保留时间相吻合(图9a)。这些结果表明,RBC-SMIPs作为固相萃取吸附剂结合高效液相色谱,实现了对牛奶中TC的高效吸附、富集与检测。

**图 9:**
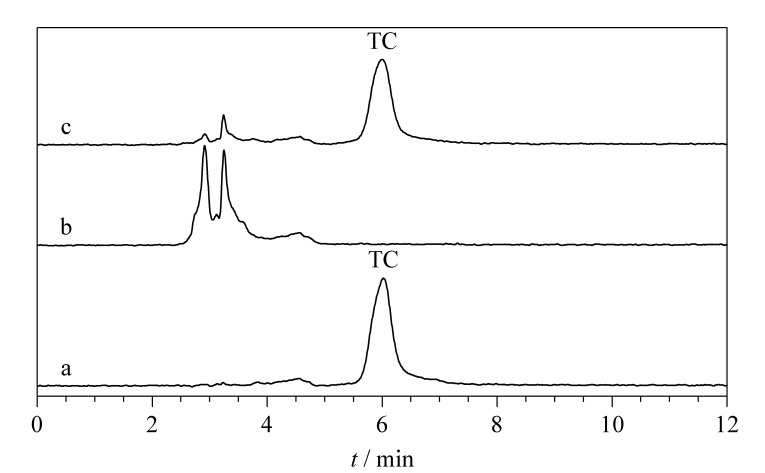
(a)TC标准溶液和加标(0.05 μg/mL)牛奶样品经RBC-SMIPs富集(b)前、(c)后的色谱图

### 2.5 与其他吸附剂比较

将本工作与其他已发表的使用MIPs作为吸附剂吸附和分离TC的研究进行了比较。由表2可知,RBC-SMIPs具有较高的吸附能力和选择性。类红细胞双凹圆碟结构可以为TC模板分子提供更大的比表面积和更易接近的识别位点,从而提高了材料的吸附容量(76.16 mg/g),比其他已报道的MIPs吸附量至少提高了1.6倍,此外,材料展现出更高的印迹因子和选择系数。

**表 2:** 与其他相关工作的比较

Adsorbents	Q_MIPs_/(mg/g)	IF	SC	Ref.
MMIP NPs	40.48	2.55	1.24-3.18	[27]
Fe_3_O_4_@MI-POSS	21.91	1.30	1.69-3.38	[28]
DA+BSA-MMIPs	38.48	2.01	1.33-1.90	[29]
TMIP	47.78	3.37	1.41-3.03	[30]
RBC-SMIPs	76.16	3.62	1.70-3.22	this work

*Q*_MIPs_: adsorption capacity of molecularly imprinted materials for TC; SC: selective factor.

## 3 结论

本工作以SiO_2_纳米球作为牺牲载体,四环素为模板分子,DA为功能单体,通过将表面分子印迹技术和牺牲载体方法相结合,合成对TC具有特异性识别能力的类红细胞状高吸附容量表面分子印迹聚合物。一系列实验表明所制备印迹材料具有较大的吸附容量、较快的吸附平衡时间、较高的选择性和优异的重复利用性。此外,本工作建立了RBC-SMIPs作为吸附剂的HPLC方法,并应用于牛奶中TC的选择性分离分析,该材料对动物源性食品中痕量抗生素的选择性分离富集具有潜在的实际应用价值。
